# A LC-MS/MS Method for Quantifying the Schisandrin B and Exploring Its Intracellular Exposure Correlating Antitumor Effect

**DOI:** 10.1155/2023/8898426

**Published:** 2023-06-06

**Authors:** Bosu Meng, Shouhong Gao, Jihui Chen, Bin Wang, Yuhui Mu, Yan Liu, Zhipeng Wang, Wansheng Chen

**Affiliations:** ^1^College of Traditional Chinese Medicine, Yunnan University of Traditional Chinese Medicine, Kunming 650500, Yunnan, China; ^2^Department of Pharmacy, Second Affiliated Hospital of Naval Medical University, Shanghai 200003, China; ^3^Department of Pharmacy, Xinhua Hospital, Affiliated to Shanghai Jiao Tong University School of Medicine, Shanghai 200092, China; ^4^School of Chemistry and Biology, Yichun College, Yichun 336000, Jiangxi, China

## Abstract

Schisandrin B (Sch.B) shows antineoplastic activity in colorectal cancer, but the mechanism is still obscure. The intracellular spatial distribution may be helpful in elucidating the mechanism. To investigate the intracellular drug distribution of Sch.B in cancer cells, a simple, rapid, and sensitive ultra-highperformance liquid chromatography tandem mass spectrometry (UHPLC-MS/MS) method was established for the determination of Sch.B in colorectal cancer cells. Warfarin was utilized as an internal standard. The sample pretreatment was carried out with protein precipitation using methanol. The analyte was separated on an Atlantis T3-C_18_ column (3 *μ*m, 2.1*∗*100 mm) using gradient elution with a mobile phase comprised of methanol and 0.2% formic acid in water. The flow rate was 0.4 mL/min. The linear range of Sch.B was 20.0–1000.0 ng/mL with a correlation coefficient (*R*) more than 0.99. The matrix effect and recovery ranged from 88.01% to 94.59% and from 85.25% to 91.71%; the interday and intraday precision and accuracy, stability, specificity, carryover, matrix effect, and recovery all conformed to the requirements of pharmacopoeia. Cell viability and apoptosis assays demonstrated that Sch.B has an inhibitory effect in a dose-dependent way on HCT116 proliferation and achieved significant suppression at 75 *μ*M (IC_50_). It was found that in HCT116 cell, nucleus, and mitochondria, exposure levels of Sch.B peaked at 36 h and then decreased, and mitochondria possessed more Sch.B than nucleus. These results may help to elucidate the antitumor effect of Sch.B.

## 1. Introduction

**Figure 1:**
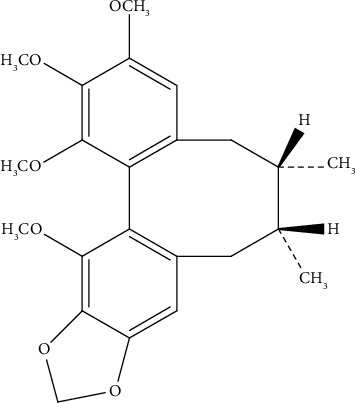
Chemical structure of Sch.B.

Colorectal cancer (CRC) is the third most common cancer and the second leading cause of cancer-related death in the developed countries. According to the global cancer statistics [1], the new cases of CRC in 2020 have reached 19.3 million with nearly 10 million deaths, which causes serious health, social, and economic problems. The occurrence of CRC is related to lifestyles and environmental and genetic factors, including alcohol consumption, smoking, lack of exercise, low fruits and vegetables intake, and high-fat and high-salt diet and so on [2]. In recent years, a part of traditional Chinese medicine has been reported to possess an anticancer effect [3], besides the chemotherapeutic drugs commonly used in clinical applications; for example, Schisandrin B (Sch.B, Figure 1), one of the bioactive components in the fruit of *Schisandra chinensis*, has been proved to exert diverse pharmacological effects including antioxidantion, neuroprotection, myocardial preservation, and liver and kidney protection [4–6]. In addition, Sch.B has antitumor activities against a various of cancers including osteosarcoma, cholangiocarcinoma, gallbladder carcinoma, gastric cancer, breast cancer, and glioma [7–12] through reversing multidrug resistance, broadly inducing apoptosis [13–16], neuroprotection [17], cell cycle arresting [18], and inhibition of proliferation, migration, and invasion [19]. Importantly, Sch.B can enhance panitumumab cytotoxicity in all CRC cell lines and promoted panitumumab-induced apoptotic cell death through caspase-3 activation, DNA fragmentation, and Bcl-2 downregulation [20]. Sch.B can further target multiple tumor-promoting signaling pathways including epithelial-mesenchymal transition [6, 19, 21] to enhance the effects of other antitumor drugs, for example, apatinib and docetaxel [22, 23]. In CRC, Sch.B could induce the SMURF2 protein expression and affected SIRT1 in vitro and in vivo, which negatively correlated each other and may be involved in the antitumor effect [24]. Sch.B has an effect on the regulation of gut microbiota and the protection of the intestinal epithelial barrier, which resulted in the inhibition of colitis-associated cancer [25]. But the underlying mechanisms and direct targets of Sch.B exerting an anticancer effect have not been elucidated, and the spatial exposure level of Sch.B in cancer cells may contribute to finding its targets and clarifying the anticancer mechanisms. Thus, the aim of this study is to develop a simple, rapid, and sensitive UHPLC-MS/MS method to profile the spatial distribution of Sch.B in the HCT116 cell line and assess the anticancer effects of Sch.B against the HCT116 cell line.

## 2. Materials and Methods

### 2.1. Chemicals and Reagents

Sch.B (purity ≥98%) and warfarin (internal standard, IS) were purchased from Tongtian Biotech Co., Ltd. (Shanghai, China). HPLC-grade methanol (MeOH) was obtained from Merck (Merck Company, Darmstadt, Germany). HPLC-grade formic acid was from McLean Biotech Co., Ltd. (McLean, Shanghai, China). Dimenthyl sulfoxide (DMSO) in analytical grade was bought from Tedia Company Inc. (Tedia, Fairfield, USA). Distilled water was obtained from Shenzhen Watsons Distilled Water Co., Ltd. (Shenzhen, China). High and low glucose cell culture mediums were purchased from Meilune Biotech Co., Ltd. (Dalian City, China). A TUNEL apoptosis assay kit was obtained from Beoytime Biotechnology (Shanghai, China). The cell nucleus and mitochondria isolation and extraction kits were obtained from Abbkine Scientific Co., Ltd. (Wuhan, China).

### 2.2. Cell Culture

Human colorectal cancer cell line HCT116 was bought from Meilune Biotech Co., Ltd. (Dalian City, China) and routinely cultured in Dulbecco's modified Eagle's medium (DMEM, Meilune, China) basic medium supplemented with 10% heat-inactivated fetal bovine serum (FBS, Thermo, America), glutamine, and 1% penicillin-streptomycin at 37°C in a 5% CO_2_ atmosphere. The medium was refreshed every 2 days before the cells reached 85%–90% confluence, and then the cells with less than 10 passages were used to perform experiment or make stock solution. The mycoplasma contamination was tested every month.

### 2.3. LC-MS/MS Instrumentation

The experiments including method development and Sch.B determination were performed on an Agilent 1290 series UHPLC system which includes an online degasser, a binary pump, an autosampler, and a column oven and interfaced to an Agilent 6460A triplequadrupole mass spectrometer with an electrospray ionization source (ESI, Agilent Technologies, USA). Data were acquired and analyzed using Agilent MassHunter data processing software (version 6.00).

### 2.4. Liquid Chromatographic Condition

The chromatographic separation of Sch.B was achieved based on an Atlantis®T3-C_18_ column (2.1*∗*100 mm, 3 *μ*m, Waters Co., Milford, USA). The mobile phase A is water (containing 0.2% formic acid), and the mobile phase B is MeOH, and the initial mobile phase consisted of 100% phase A and the gradient elution program were: 0 min, 0% B; 0–0.5 min, 0% to 90% B; and then increased to 100% phase B at 0.51 min until 4 min. The total running time was 4 min and the post-time was 2 min. The injection volume was 5 *μ*L with column temperature kept at 25°C.

### 2.5. Mass Spectrometry Condition

**Figure 2:**
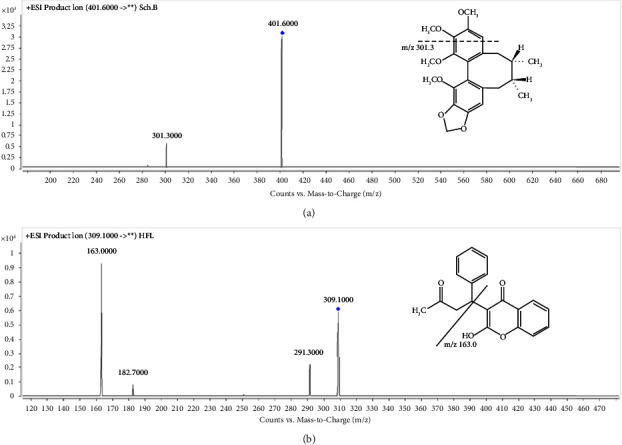
Product ions chromatograms and fragment structures of Sch.B (a) and IS (b).

Ionization of the analyte was accomplished in an ESI source operating in the positive ionization mode with the capillary voltage set at 4500 V. All data collection was carried out in multiple reaction monitoring (MRM) mode (Figure 2). Nitrogen was used as dry gas, sheath gas, and nebulizer gas. The nebulizer pressure was 45 psi. The temperature of sheath gas was set at 300°C, and the flow rate was 12 L/min. The drying gas was heated to 320°C and delivered at 10 L/min. High purity nitrogen, which was set at 0.2 MPa, was used as collision gas. The mass spectrometry parameters of Sch.B and IS are listed in Table 1.

### 2.6. Preparation of Standard Solution and Quality Control Sample

A 2.0 mg of Sch.B was accurately weighted and then was added with an appropriate amount of MeOH to obtain the stock solution at the concentration of 1.0 mg/mL, and then the stock solution was aliquoted in 1.5 ml tubes and stored at −80°C until use. The stock solution of Sch.B was diluted with MeOH to get a series of working solutions, and then the working solutions were diluted with blank medium (DMEM) to obtain calibration standards at the following concentrations: 20.0, 50.0, 100.0, 200.0, 500.0, 750.0, and 1000.0 ng/mL. Quality control (QC) samples were prepared separately using the same method at concentrations of 20.0, 50.0, 200.0, and 750.0 ng/mL and stored at −80°C. The IS stock solution with concentration at 1.0 mg/mL was prepared in MeOH and stored at −80°C. And the IS work solution was diluted freshly with MeOH to gain finally 100 ng/mL when needed.

### 2.7. Cell Sample Collection and Sample Pretreatment

A total of 1*∗*10^6^ HCT116 cells per well were grown in 6-well plates in triplicate for 24 h. After the confluence reached 80–90%, medium was refreshed with 0.1% BSA low-glucose DMEM containing 75 *μ*M Sch.B for 12 h, 24 h, 36 h, and 48 h. Then the treated cells were cleaved by adding 1 mL MeOH and placed for 10 min after washing 2 times with precooled PBS. Cell nucleus and mitochondria were harvested using ExKine™ nuclei and mitochondrion extraction kit (High Purity). A 100 *μ*L aliquot of cell lysis sample was mixed with MeOH 900 *μ*L for 2 min, and then 100 *μ*L of the mixture was taken to whirl and centrifuge (14500 × *g*, 10 min) after adding 300 *μ*L MeOH (containing 100 ng/ml IS), and then a 100 *μ*L supernatant was transferred to the sample vial for analysis. For the cell nuclear and mitochondrial extraction, the ExKine™ nuclei and mitochondrion extraction kit (High Purity) was used and a 100 *μ*L of extract was added with 300 *μ*L MeOH (containing 100 ng/ml IS) before a 2 min's vortex and 14500 × *g* centrifugation for 10 min, and the supernatant was injected into the LC-MS/MS system. All experiments were performed in triplicate.

### 2.8. Cell Viability, Apoptosis, and Colony Formation Assays

A total of 5*∗*10^3^ HCT116 cells per well were grown in a 96-well plate overnight. Cells were treated with 0.1% BSA low-glucose DMEM containing Sch.B (9.375, 18.75, 37.5, 75, and 150 *μ*M) for 24 h and 48 h. The CCK-8 kit was used according to the instructions to detect cell proliferation after pretreatment. Finally, the absorbance was measured at 450 nm using a SYNERGY microplate reader (BioTek, Winooski, USA), and then the formula ((OD treatment − OD blank)/(OD control − OD blank) × 100) was applied to calculate cell viability. The apoptosis assay was used to evaluate the effect of Sch.B on the proliferation of HCT116 cells. The cell apoptosis status of HCT116 cells treated with Sch.B was analyzed by a fluorescent inverted microscope. A TUNEL assay was carried out as follows: first, cells were plated on coverslips at the bottom of 12-well plate, and then HCT116 cells were seeded at 1.2 *∗* 10^5^ per well in 12-well plate for 24 h and then treated with a series of concentrations of Sch.B for another 24 h. The cells were incubated according to the manufacturer's instruction. Images were captured after PBS washing.

### 2.9. Methodological Study

The method validation was completed according to FDA guidelines and the Chinese Pharmacopoeia (2020 edition), and the linearity, interday and intraday precision and accuracy, matrix effect and extraction recovery, carryover, stability, and specificity of this method were validated according to previous study [26–28].

### 2.10. Data Analysis

The raw data were analyzed using GraphPad Prism (GraphPad Software, Chicago, USA, version 7.01) and SPSS software (IBM Corporation, USA, version 19.0.0). Comparisons between groups were analyzed using a one-way ANOVA or the Student *t* test. *P* value <0.05 was considered to have statistically significant.

## 3. Results and Discussion

### 3.1. LC-MS/MS Optimization

To find better parameters for analyte detection during the method development, the ionization efficiency of Sch.B in positive and negative ionization modes were first evaluated, and the results found that positive ionization mode had a higher response of Sch.B than negative ionization mode. It was found that the Sch.B has better peak shape, response, and suitable retention time in the Atlantis T3-C_18_ column after testing different columns. Furthermore, the influence of column temperatures (25°C, 30°C, 35°C, and 40°C) on the retention time of the Sch.B was considered, and lower temperature was more suitable for the Sch.B retention than higher temperature, and the retention time was optimized to 2 min. Besides, the mobile phase containing various additives, for instance, ammonium acetate, formic acid and ammonium formate in methanol, acetonitrile, or water was tested and the results showed that methanol produced strong signals, and then a 0.2% formic acid in water increased the efficiency of ionization, giving better peak shape and response of Sch.B and reduced matrix effects.

### 3.2. Sample Extraction

Sample pretreatment is a critical step in the removal of matrix interferents, which can get higher recovery and more steady matrix effect. Different methods containing protein precipitation, solid-phase extraction, and liquid-liquid extraction to remove protein and other interferents in the matrix were evaluated. It was found that solid-phase extraction and liquid-liquid extraction yielded low recoveries for Sch.B (both <40%). In the process of protein precipitation, methanol showed higher recovery (>80%) and more stable matrix effect than acetonitrile. Finally, by comparing the recovery and matrix effect of methanol as precipitant in different proportions, the ratio of sample to methanol was selected as 1 : 3 (V : V), obtaining better recovery and matrix effect.

### 3.3. Method Validation

#### 3.3.1. Specificity

**Figure 3:**
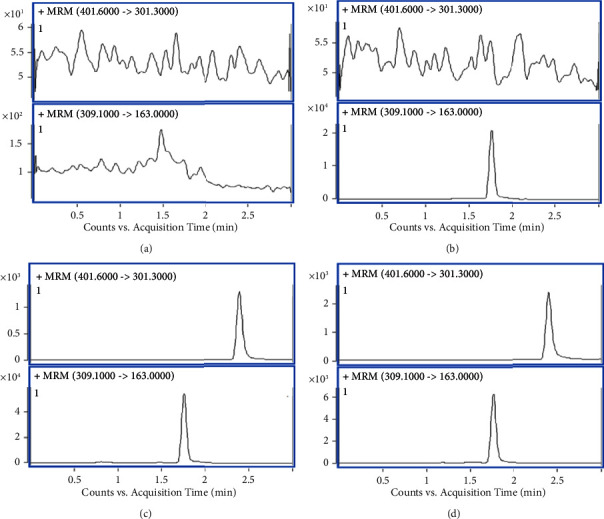
Representative MRM chromatograms of the specificity of Sch.B. (a) Blank sample; (b) blank sample spiked with IS; (c) LLOQ sample; (d) real sample.

The retention times for Sch.B and IS were 2.43 min and 1.76 min, respectively. In order to evaluate the specificity, the responses from blank sample, IS spiked sample, LLOQ sample, and real sample in six different lots were compared (Figure 3). The result showed no significant interferences as the responses in the blank sample were less than 20% of Sch.B in the LLOQ sample and 5% of IS.

#### 3.3.2. Linearity of Calibration Curves and Lower Limited of Quantification (LLOQ)

Calibration curves were established by calculating the peak area ratio (analyte/IS) of Sch.B with IS versus Sch.B nominal concentrations. The calibration range of Sch.B was from 20 to 1000 ng/mL, and seven calibration standards were prepared in this range, and the best linearity and least squares were obtained using a 1/*χ*^2^ weighing factor. The linear correlation coefficient *R*^2^ is more than 0.99 for Sch.B. The typical regression equation is *Y* = 0.008950*∗x*+0.006233, *R* = 0.9986, and LLOQ = 20 ng/mL. The LLOQ is the lowest concentration point in calibration curve that can be in accordance with the accuracy within ±20% and precision ≤20%. The results are listed in Table 2.

#### 3.3.3. Interday and Intraday Precision and Accuracy

The interday and intraday precision and accuracy of the method were assessed in LLOQ, low, middle, and high QC samples (*n* = 5). Interday and intraday precision and accuracy for Sch.B are summarized in Table 3. The interday precision and intraday precision (RSD%) were 1.51%–5.28% and 1.56%–5.4% for Sch.B, and the interday accuracy and intraday accuracy (RE%) ranged from 1.83% to 4.03% and from 2.33% to 3.93%. The data meet the requirements of pharmacopoeia.

#### 3.3.4. Matrix Effect and Extraction Recovery

The results of recovery and matrix effect are presented in Table 4. The low, middle, and high QC samples (50, 200, and 750 ng/mL) in three replicates were investigated. It was found that the matrix effect and extraction recovery of Sch.B were in the range of 88.01%–94.59% and 85.25%–91.71%. The RSD% of matrix effect and extraction recovery were 0.89%–7.32% and 1.65%–8.75%, which were less than 15% and meet the requirements.

#### 3.3.5. Stability

The results of the stability are shown in Table 5. The long-term stability, short-term stability, and freeze-thaw stability of Sch.B in DMEM were investigated. Long-term stability and short-term stability were defined as the stability of the QC sample after two months in a refrigerator at −80°C and in the autosampler at 4°C for 12 h; freeze-thaw stability was evaluated using the QC sample after three freeze-thaw cycles (−80°C to room temperature). The deviations meet the requirements (RE within ±15%) for the analyte.

### 3.4. Sch.B Inhibited Proliferation of the HCT116 Cell

**Figure 4:**
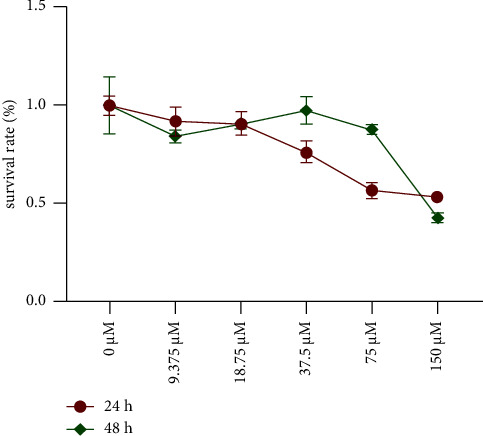
Cell viability of HCT116 cells treated with different concentrations of Sch.B.

The HCT116 cytotoxicity induced by Sch.B was investigated using the CCK-8 assay. The cells were treated with a series of concentrations of Sch.B (9.375, 18.75, 37.5, 75, and 150 *μ*M) for 24 h and 48 h (Figure 4). The experiment results showed a dose-dependent inhibitory effect on the cell growth of HCT116. According to the previous experiments and summary, the median inhibitory concentration of Sch.B (75 *μ*M) was selected for further cell experiments.

### 3.5. Sch.B Promoted Cell Apoptosis of HCT116

**Figure 5:**
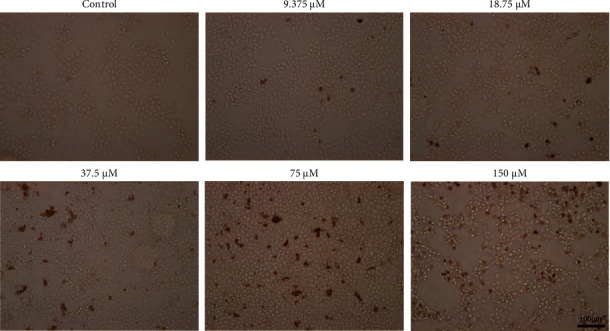
Results of TUNEL apoptosis experiment treated with Sch.B in different concentrations.

In order to investigate the effect of Sch.B on HCT116 cell apoptosis, a TUNEL assay was carried out. The cells were treated for 24 h with a series of concentrations of Sch.B (0, 9.375, 18.75, 37.5, 75, and 150 *μ*M). The brown fluorescence intensity indicated the number of apoptotic HCT116 cells. As shown in Figure 5, the results found that the inhibitory of Sch.B on HCT116 proliferation exerted in adose-dependent way and achieved a significant effect at 75 *μ*M. Sch.B can suppress proliferation and promote HCT116 cell apoptosis.

### 3.6. The Spatial Exposure Levels of Sch.B in HCT116 Cells, Nucleus, and Mitochondria

**Figure 6:**
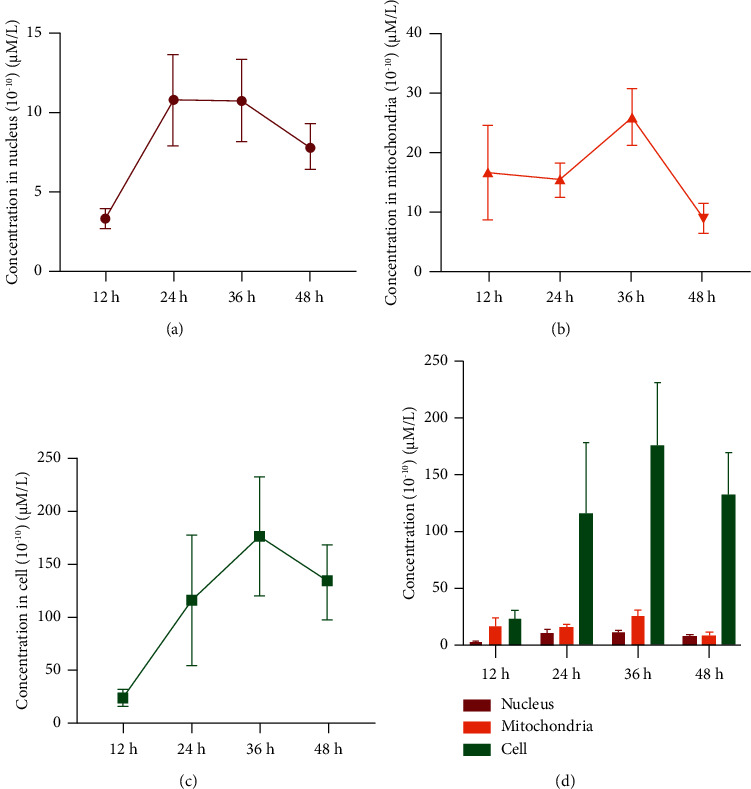
Time and spatial variation of Sch.B exposure in nucleus, mitochondria, and cells at 12, 24, 36, and 48 h. A parallel experiment was carried out to measure the cell number and normalize the concentration of Sch.B in single cell (*n* = 3). (a) The concentration-time curve of Sch.B in the nucleus of HCT116 cells. (b) The concentration-time curve of Sch.B in the mitochondria of HCT116 cells. (c) The concentration-time curve of Sch.B in the HCT116 cells. (d) The comparative results of concentrations of Sch.B in HCT116 cells.

To further investigate the ways that Sch.B inhibits HCT116 cancer cell, this study determined the intracellularly spatial exposure levels of Sch.B in the HCT116 cells, nucleus, and mitochondria. The measured results of intracellular samples, mitochondria samples, and nucleus samples are exhibited in Figure 6. It was found that higher concentration of drug entered cells and nucleus with the prolongation of drug treatment time and increase of concentrations, but the exposure level of Sch.B in cell, nucleus, and mitochondria decreased after 36 h, which may be attributed to the efflux transport. Sch.B has a core structure of dibenzocyclooctadiene, which inhibited efflux of chemotherapeutic drug and restored the intracellular drug accumulation by inhibiting P-glycoprotein and mutidrug resistance protein 1 [29, 30]. In addition, Sch.B was reported to protect myocardial cell through interacting with the TIR domain of MyD88 in the cytoplasm and inhibiting MyD88-independent inflammation to attenuate diabetic cardiomyopathy [31]. The spatial distribution of Sch.B may have a critical role in exerting its effects, and further studies are needed to connect the spatial exposure levels of Sch.B inside the cells with the anticancer effect.

## 4. Conclusion

A simple, rapid, and sensitive UHPLC-MS/MS method was developed and validated to measure the concentration of Sch.B in cells. The sample pretreatment method was finished using protein precipitation and the running time was 4 min. The results indicated that Sch.B can inhibit cell growth and induce apoptosis of HCT116 cells in a dose-dependent way. The spatial distribution of Sch.B in cells showed region and time discrepancy, which may be helpful in investigating the effects of Sch.B on cancer cells.

## Figures and Tables

**Table 1:** Mass spectrometry parameters of analyte and IS.

Analytes	MRM transition (*m*/*z*) (*Q*1 ⟶ *Q*3)	Fragmentor (V)	CE (eV)	Retention time (min)
Sch.B	401.6 ⟶ 301.3	135	23	2.43
IS	309.1 ⟶ 163	90	12	1.76

**Table 2:** The regression parameters of calibration curves of Sch.B.

Analyte	Regression type	Linear range	Weighing factor	Regression equations	*R* ^2^
Sch.B	Linearity	20–1000 ng/mL	1/*χ*^*2*^	*Y* = 0.008950^*∗*^*x*+0.006233	0.9986

*R*: the correlation coefficient.

**Table 3:** Interday and intraday precision and accuracy of Sch.B (*n* *=* 5).

Analyte	Nominal concentration (ng/mL)	Interday measured concentration (ng/mL) ± SD	RSD (%)	RE (%)	Intraday measured concentration (ng/mL) ± SD	RSD (%)	RE (%)
Sch.B	20	20.75 ± 1.09	5.28	3.77	20.49 ± 1.10	5.40	2.49
	50	52.01 ± 2.34	4.50	4.03	51.94 ± 2.05	3.95	3.89
	200	207.43 ± 9.20	4.43	3.71	207.87 ± 8.24	3.96	3.93
	750	763.73 ± 11.54	1.51	1.83	767.54 ± 12.00	1.56	2.33

**Table 4:** Extraction recovery and matrix effect of Sch.B.

Analyte	Nominal concentration (ng/mL)	Recovery		Matrix effects	
		Mean (%) ± SD	RSD (%)	Mean (%) ± SD	RSD (%)
Sch.B	50	91.71 ± 8.02	8.75	88.01 ± 0.78	0.89
	200	85.25 ± 1.40	1.65	94.59 ± 5.62	5.94
	750	88.61 ± 1.79	2.02	91.88 ± 6.73	7.32

**Table 5:** The stability of Sch.B in different conditions (*n* = 5).

Analyte	Nominal concentration (ng/mL)	Freeze-thaw stability measured concentration (ng/mL)	RSD (%)	Short-term stability measured concentration (ng/mL)	RSD (%)	Long-term stability measured concentration (ng/mL)	RSD (%)
Sch.B	50	53.82	5.62	54.26	8.40	56.20	6.18
	200	211.07	6.35	203.19	3.10	210.22	1.10
	750	749.98	12.81	757.04	2.00	754.87	1.71

## Data Availability

The data used to support the findings of this study are available from the corresponding author upon request.
